# Mediterranean Diet and the Emotional Well-Being of Students of the Campus of Melilla (University of Granada)

**DOI:** 10.3390/nu12061826

**Published:** 2020-06-19

**Authors:** María López-Olivares, Miriam Mohatar-Barba, Elisabet Fernández-Gómez, Carmen Enrique-Mirón

**Affiliations:** 1 Doctoral Degree School, Melilla Campus, University of Granada, Calle Santander s/n, 52001 Melilla, Spain; marialopez93@correo.ugr.es; 2 Department of Nursing, Faculty of Health Sciences, Melilla Campus, University of Granada, Calle Santander s/n, 52001 Melilla, Spain; miriamb@ugr.es; 3 Department of Inorganic Chemistry, HUM-613 Research Group, Faculty of Health Sciences, Melilla Campus, University of Granada, Calle Santander s/n, 52001 Melilla, Spain; cenrique@ugr.es

**Keywords:** healthy diet, positive affect, negative affect, state of health, healthy behavior

## Abstract

A certain link exists between the consumption of particular groups of food and well-being. In this study, we analyzed in depth the relationship between strict adherence to the Mediterranean diet (MD) and emotional well-being through a descriptive, exploratory, transversal, and correlational study of students from the Campus of Melilla, University of Granada, Spain. The sample consisted of 272 individuals. Adherence to the MD was measured with the PREvención con DIetaMEDiterránea (PREDIMED) questionnaire, emotional well-being (both positive and negative affection) with the Spanish version of the Positive and Negative Affect Schedule (PANAS), the state of perceived health with the Short Form-36 (SF36), and the degree of physical activity using the International Physical Activity Questionnaire Short Form (IPAQ-SF). A strict adherence to the MD was found to be significantly related to positive emotional state (β = 0.018, *p* = 0.009). The perceived state of health (β = 0.192, *p* < 0.001), mental role (β = 0.346, *p* < 0.001), and physical activity (β = 0.155, *p* = 0.007) were found to be predictive factors of a positive emotional state. Conversely, the relationship between the adherence to the MD and a negative emotional state was not significant. Various components of the MD were found to be independently connected to well-being. The results suggest that adopting a nutritional pattern such as the MD is linked to an improvement in emotional well-being.

## 1. Introduction

The age range considered to correspond to adolescence varies. Some authors delimit adolescence as the period between 10 and 20 years of age [1], but The Lancet commission about health and well-being divides adolescence into three categories: early adolescence (10–14 years), late adolescence (15–19 years), and young adulthood (20–24 years). Physiologically, early adolescence is dominated by puberty and sexual development; late adolescence is characterized by pubertal maturation, although to a lesser extent than early adolescence; and young adulthood corresponds to the adoption of the roles and responsibilities of adults [2]. Adolescence is, in general, a period of physical development characterized by significant changes in the cognitive, psychological, and emotional dimensions, which affect the quality of life (QoL), well-being, and state of health of teenagers [3,4]. Specifically, this phase of development is one of the most complex, since this is when unhealthy behaviors can be acquired [1,5]. Students are young adults whose first university years represent a period of vulnerability, in which the prevalence of symptoms such as depression and anxiety are steadily growing [6]. The early university years are an important stage for the acquisition of behavioral habits [7,8,9,10], as many students leave their family homes to start their university studies and to live independently [9,10].

A healthy lifestyle is composed of a set of factors that must work harmoniously and completely. Some variables demonstrated by research to be the most influential in a healthy lifestyle are eating habits, physical activity, physical inactivity, and emotions [11]. A healthy lifestyle is a key component in fostering healthy aging and in reducing the risk of cardiovascular events, which requires, among other aspects, participating in regular physical activity, reducing sedentary behavior, and maintaining a good quality of life [12]. Some studies [13,14,15] have connected the impact of physical activity and physical inactivity with quality of life.

Concerns about nutrition issues in adolescents are growing [16,17]. The eating habits and nutritional knowledge acquired during this stage are key factors for the creation of good eating habits in adulthood [18]. The management of a healthy diet during adolescence can positively affect psychological well-being and the maturation of the brain, since the most important brain structures develop quickly during this period [18,19,20]. Therefore, improving the comprehension of eating habits and patterns of healthy lifestyles during this period can help to identify factors that foster good health [18].

The term “Mediterranean diet” (MD) is used to describe the traditional eating habits of people that live near the Mediterranean Sea, especially in the areas of olive cultivation. The MD is defined by the frequent consumption of fruits, vegetables, whole grains, legumes, nuts, and seeds, with olive oil as the main source of added fat. Other characteristics of the MD are the regular and moderate intake of dairy products, the moderate intake of fish and poultry, limited red meat, and moderate consumption of wine [18,21,22,23]. These foods play an important role in the prevention of cardiovascular diseases, cerebrovascular diseases, diabetes, obesity, and neurodegenerative diseases [24,25,26], providing benefits against cognitive impairments, including Alzheimer’s disease [27], and cancers [24,25,26]—specifically bowel and breast cancers [28].

An important factor that can influence food consumption is emotions. Emotions are regulated by a range of procedures such as the intensity, duration, and the type of emotion experienced. Emotional regulation has been connected to a wide variety of functioning domains, including social functioning, psychological and physical well-being, and academic performance. Despite the importance of the regulation of emotions in daily life, little is known about their development. However, adolescence is relevant for the regulation of emotions, since it is a period during which quick and fundamental alterations occur in adolescents’ emotional states [29].

Emotional intelligence (EI) has become the focus of a number of researchers. EI is understood as the ability to adequately perceive, understand, and develop emotions. This construct represents one of the most relevant elements during the period of secondary and higher (i.e., university) education [30,31]. Various studies [32,33] have proved that EI is crucial in emotional regulation and in the control of feelings in educational environments. EI also facilitates psychological well-being, reduces negative emotions, and fosters the creation of healthy behaviors. Low levels of EI directly impact the creation of bad habits, such as inactive lifestyles, following a diet with low adherence (e.g., to the MD), or tobacco and alcohol consumption [30,31]. Therefore, certain motivational and emotional environments that help form healthy habits and reduce harmful conducts must be fostered [31]. 

Emotional intake derived from eating is a response to affective states. Emotions strongly influence the choice of food and eating habits [34,35], and therefore, many people eat as a response to a variety of negative emotions, such as anxiety, depression, and anger. Emotional eating has been connected to excess intake, binge eating, bulimia nervosa, and obesity [34]. According to psychosomatic theory, people that ingest food depending on their emotions do not have the ability to discern between hunger and negative emotional states [35]. However, a high adherence to the MD was shown to be connected with greater happiness in adolescents, specifically in psychological well-being, better mood, and better financial and social resources [18].

The main objective of this study was to analyze the relationship between adherence to the MD and the emotional state of Spanish university students at the Campus of Melilla (University of Granada), together with other variables such as state of health and degree of physical activity.

## 2. Materials and Methods 

### 2.1. Study Design and Participants 

This study was descriptive and exploratory, of the transversal and observational type, using a descriptive–correlational research methodology.

The sample was selected by chance or by incidental non-probability sampling, with the population being university students from the Melilla Campus (University of Granada). Once provided with details during an information session, a total of 272 subjects from the Campus of Melilla agreed to participate in the study, whose average age was 20.97 ± 3.61 years. The characteristics of the sample regarding sex, studies, place of origin, and religion are shown in Table 1.

### 2.2. Instruments and Procedure

To assess adherence to the MD, the 14-point questionnaire used in the PREvención con DIetaMEDiterránea (PREDIMED) study [36]—which is a nutritional intervention study that evaluated the efficacy of the MD in the long-term prevention of cardiovascular diseases—validated in a similar cohort in Spain was used [37]. Higher scores in this questionnaire indicate stricter adherence to the MD; thus, scores equal to or greater than 9 indicate strict adherence to the MD, whereas scores lower than 9 indicate low adherence to the MD.

Emotional state (both positive and negative affect) was determined through the Spanish version of the Positive and Negative Affect Schedule (PANAS) questionnaire, which is composed of 20 items, 10 of which refer to the positive affect (PA) subscale (i.e., interested, cheerful or thrilled, energetic, excited, proud or satisfied, willing or free, inspired, determined or daring, attentive or thorough, and active) and the other 10 items refer to the negative affect (NA) subscale (i.e., tense or stressed, upset or annoyed, guilty, scared, angry or furious, irritable or grumpy, embarrassed, nervous, restless, or worried, and scared or afraid). The measurement scale ranges between 0 and 50 in both cases. Larger values (closer to 50) for the PA are positive, while for the NA, the opposite is true.

For the variables related to the state of health, the Short Form-36 (SF36) (version 2) in Spanish was employed, which consists of 36 items that value the quality of life related to health, grouped into eight subscales or dimensions: physical functioning (FF), physical role (RF), body pain (DC), general health (SG), vitality (VT), social functioning (FS), emotional role (RE), and mental health (SM). This questionnaire counts each item to determine the general perceived state of health and allows for the calculation of two total components based on two subscales, namely, physical (FF + RF + DC + SG) and mental (RE + FS + SM + VT) [38].

Finally, the degree of physical activity (i.e., low, moderate, and high) was determined through the International Physical Activity Questionnaire Short Form (IPAQ-SF) questionnaire [39], with which the time (in minutes) and the frequency (in days) dedicated to activities of different intensities were collected. 

The participants completed the questionnaires in-person after written informed consent was provided. The data were collected between October and December of 2019.

### 2.3. Statistical Analysis 

Descriptive statistics were used (i.e., frequencies and percentages) to describe the characteristics of the sample. The averages and the percentages of the state of health were calculated, as well as the physical and mental components, the well-being variables (i.e., PA and AN), and the adherence to the MD. Two multiple regression models were built for each variable. The first one included the average score obtained using PREDIMED, and the second included each of its 14 items. All of the models simultaneously considered age, sex, physical activity, general state of health (i.e., RF and RM), and self-perceived state of health as possible confounding factors. Estimation by ordinary least squares was employed, which has been proved to be robust to deviations from normality in large enough samples.

In all the regression models, non-standardized and standardized regression coefficients (β) were calculated, which are a measure of the size of the effect linked to the non-standardized regression coefficients.

All the analyses were calculated with IBM SPSS Statistics for Windows, Version 24.0 (International Business Machines Corporation (IBM), Armonk, NY, USA).

### 2.4. Ethics

This research was conducted in compliance with the ethical principles set out in the Declaration of Helsinki. All participants were informed of the purpose of this study and participated voluntarily, having signed an informed consent form. The study received approval by the management of the Campus of Melilla.

## 3. Results

Table 2 shows the overall profile of the sample, including different aspects of health, healthy habits (i.e., physical activity and adherence to the MD), and emotional well-being.

Table 3 lists the frequencies of the positive scores for each one of the items of PREDIMED, as well as the total average score of the sample (8.59 ± 1.98). A total of 10 of the 14 items of the questionnaire were scored positively by more than half of the sample. Conversely, less than half of the participants ate at least three portions of fruit per day (34.80%), drank three or more glasses of wine per week (5.10%), consumed three portions of legumes per week (48.70%), or ate three portions of fish or seafood per week (49.50%). Most of the sample responded that they used olive oil as their main culinary fat (95.20%).

The results of the regression models with each one of the well-being measures as the dependent variables and the PREDIMED score as the independent variable are shown in Table 4. The overall score was found to be significantly associated with PA (β = 0.018, *p* = 0.009). Perceived health state (β = 0.192, *p* < 0.001), mental role (β = 0.346, *p* < 0.001), and physical activity (β = 0.155, *p* = 0.007) were found to be predictive factors of positive emotional state. Conversely, the relation of overall MD adherence score with NA was not significant. The perceived state of health (β = 0.189, *p* = 0.008) and mental role (β = 0.502, *p* < 0.001) showed an inverse significant relationship with negative affect.

Table 5 lists the results of the regressions with the different elements of the PREDIMED questionnaire as independent variables. PA showed direct significant relationships with the consumption of fruit (β = 0.010, *p* = 0.005), legumes (β = 0.203, *p* = 0.032), and sauté (β = 0.069, *p* = 0.020) (with a sauce including tomato and onion, leek, or garlic and slow-cooked with olive oil). We found a significant inverse relationship with the low consumption of red meat, burgers, or other meat products (β = 0.055, *p* = 0.023) and PA.

A low intake of commercial sweets or cakes had a direct and significant relationship with NA (β = 0.080, *p* = 0.002). The relationships regarding the preference for the consumption of white meats such as poultry or rabbit instead of red meat (β = 0.029, *p* = 0.028), and the consumption of nuts (β = 0.086, *p* = 0.042) with NA was also direct and significant. The low consumption of butter, margarine, and cream was linked inversely to NA (β = 0.066, *p* < 0.001), and to the use of sauté to create flavor (β = 0.054, *p* = 0.005).

## 4. Discussion

The results revealed statistically significant relationships between adherence to the MD and a positive emotional state, which suggests that adopting this dietary pattern is connected with a better emotional state. Strict MD adherence is related to a slower rate of cognitive impairment [40]. Such adherence may be an indicator of the high consumption of olive oil, fish, fruits, vegetables, and legumes, which indicate a lower probability of having depressive symptoms, stress, and lower resilience [41,42,43,44,45,46,47]. The intake of olive oil is linked to a lower probability of cognitive impairment in visual memory and in improvement in verbal fluency. However, the omega-3 fatty acids, docosahexaenoic acid (DHA), and eicosapentaenoic acid (EPA) present in fish oil, which play fundamental roles in brain functioning, produce better benefits than the oleic acid, linoleic acid, and palmitic acid present in olive oil [48]. The relationship between the overall score of MD adherence and negative emotional state was not significant. The perceived state of health and the mental component showed a statistically significant inverse relationship with negative affect.

Similar results were reported by Moreno-Agostino et al. [49], although they found statistically significant inverse relationships between adherence to the MD and negative affect. Therefore, the MD is related to emotional improvements in both positive and negative aspects.

We found that a stricter adherence to the MD is linked to higher scores in the mental component of quality of life, suggesting a mental/emotional benefit of this dietary pattern [50]. Similar results were reported between a stricter adherence to the MD and a better quality of life and each one of its dimensions [51].

We observed that the consumption of some components of the MD was correlated with different dimensions of well-being. Regarding PA, direct significant relationships were found with the consumption of fruit, legumes, and with the use of sauté. This means that the greater the consumption of these foods, the better the positive affect of the sample. According to some authors [41,42,43,44], consumption of fruits, vegetables, and legumes are related to lower levels of stress and greater resilience. An increase in the consumption of these foods produces an improvement in inflammation and physiological processes, which are closely linked to well-being and a lower risk of depression [52,53,54]. Ferrer-Casales et al. [18] found that omega-3 fatty acids, present in fish, nuts, and grains and the group of B vitamins found in fruits and vegetables are the most important nutrients for the functioning of the central nervous system, such as for neurotransmission, for gene expression, and for an adequate mood.

Regarding the consumption of red meat, burgers, or other meat products such as ham or sausages, a significant inverse relationship was found with PA. However, a reduction in the intake of red meat did not improve emotional well-being in an inconclusive study, so more research is needed to determine if these parameters influence the long-term perceived quality of life, mood, and sleeping patterns [55]. Although a reduction in the intake of red meat is necessary to reduce the risk of chronic diseases, it may not be the most effective strategy to achieve improvements in mental health, such as depression or anxiety [56,57].

The low intake of commercial sweets or cakes was found to have a direct and significant relationship with negative affect. Some studies ratified that the consumption of commercial sweets or cakes results in episodes of anxiety and emotional stress [58,59]. Likewise, studies have demonstrated that the glucose present in sugar and, consequently, in commercial sweets and cakes has a flattering effect on memory tasks [60]; meanwhile, other studies have concluded that a lower consumption of these foods is related to a higher possibility of suffering psychological disorders [61,62].

The relationship between the preference for the consumption of white meats, such as poultry or rabbit, instead of red meat or the consumption of nuts and negative affect was also direct and significant; the greater the consumption of these foods, the lower the negative affect. The high consumption of meat, particularly red and processed meat, is related to a higher risk of cerebrovascular accidents. On the contrary, the consumption of white meat is associated with a lower risk [63].

Nuts are an important source of vitamins and phenolic compounds, as their bioavailability remains after their consumption, providing a perceptible antioxidant loading [64]. Scientific evidence has demonstrated that a high consumption of nuts is associated with late aging, as well as positively associated with cardiovascular diseases, cancer, and cognitive problems [65]. In particular, an intervention program based on the MD showed that the consumption of nuts improves attention skills, memory, abstract thinking, spatial vision, and numeracy [66]. The low consumption of butter, margarine, or cream, according to our results, is inversely associated with negative affect and with the use of sauté to flavor.

The results of this study must be interpreted considering some limitations. Firstly, the sample object of the study was limited; more studies that investigate these associations in a more representative sample are required. Some of the associations found may change when evaluating the specific foods that form these elements separately [48]. Currently, the existing evidence regarding these findings is limited, and thus, further studies on this topic are needed. Finally, the adaptability of the instruments must be improved.

Although studies have addressed the association between adherence to the MD and depression, between adherence to the MD and different measures of quality of life, and between specific food groups and well-being, the current study is one of the first to directly examine possible relationships between adherence to the MD and emotional well-being.

## 5. Conclusions

The results obtained in this study emphasize that adherence to the MD, rather than only the consumption of its isolated components, is associated with emotional well-being. The findings showed that the consumption of certain unhealthy food groups, such as commercial sweets or cakes and red and processed meats, is also correlated with well-being, which provides possible explanations for the consumption of these food groups despite their known unfavorable implications for health. However, further research is necessary to clarify the nature of these relationships, as well as their possible implications for health. The results presented in this study necessitate future investigations into whether adherence to the MD is not only correlated with but also a causal variable for lower NA and greater well-being. If so, the recommendation of MD adherence (and monitoring thereof) could provide an approach to improve the affective experience and the evaluation of the quality life of the population, which would provide benefits beyond well-being.

## Figures and Tables

**Table 1:** Characteristics of the study sample (*N* = 272).

Variable	Frequency	(%)
Sex		
Male	96	35.30
Female	176	64.70
Study		
Health Science	169	61.90
Educational Science	103	38.10
Place of origin		
Melilla	148	54.20
Other Spanish territory	124	45.80
Religion		
Christian	128	46.90
Islamic	67	24.50
Other	77	28.60

**Table 2:** Profile of the sample in terms of state of health, healthy habits (i.e., physical activity and adherence to the MD), and emotional well-being.

	Average	SD
Perceived state of health	3.29	0.86
Physical component	72.97	7.98
Mental component	53.20	7.83
Emotional state		
Positive affect	31.54	8.24
Negative affect	16.74	6.21
	**Frequency**	**%**
Physical activity		
High	163	59.70
Moderate	61	22.50
Low	48	17.80
Adherence MD		
High (PREDIMED score ≥9)	140	51.50
Low (PREDIMED score <9)	132	48.50

MD, Mediterranean diet; SD, standard deviation, PREDIMED, PREvención con DIetaMEDiterránea.

**Table 3:** Compliance with the recommendations of the MD (*N* = 272): Frequencies and percentages of affirmative responses.

Recommendations of the MD	Frequency	(%)
Use of olive oil as the main source of fat	260	95.20
Consumption of ≥4 spoons (20 mL) of olive per day	233	85.30
Consumption of ≥2 portions (100 g) of vegetables per day	156	57.10
Consumption of ≥3 portions (pieces) of fruit per day	95	34.80
Consumption of <1 portion (100–150 g) of red meat, burgers, sausages, or cold meat per day	173	63.40
Consumption of <1 portion (12 g) of butter, margarine, or cream per day	183	67.00
Consumption of <1 portion of soft or sugary drinks per day	174	63.70
Consumption of ≥o portion of wine	14	5.10
Consumption of ≥3 portions (150 g) of legumes per week	133	48.7
Consumption of ≥3 portions (100–150 g) of fish or seafood per week	135	49.50
Consumption of <3 portions of industrial pastry per week	167	61.20
Consumption of ≥3 portions (30 g) of nuts per week	165	60.40
Preference of chicken meat, turkey, or rabbit to beef, pork, burgers, or sausages (100–150 g)	241	88.3
Consumption of ≥2 portions of whole grains (with pasta, rice, or sauté meals) per week	216	79.10
	**Average**	**SD**
**PREDIMED Score**	8.59	1.98

**Table 4:** Multiple linear regression models used to evaluate the relationship between the adherence to the MD and emotional well-being.

Variable	Positive Affect (PA)			Negative Affect (NA)		
	B	SE	Β	B	SE	Β
Overall score of adherence to the MD	0.029 **	0.036	0.018	−0.140	0.037	−0.065
Sex (ref. = male)	0.457	0.994	0.027	0.636	0.753	0.049
Age	−0.009	0.123	−0.004	0.026	0.166	−0.073
State of perceived health	1.842 ***	0.680	0.192	−1.371 **	0.514	−0.189
Physical role	−0.036	0.076	0.192	0.104	0.057	0.134
Mental role	0.362 ***	0.066	0.346	−0.400 ***	0.051	−0.502
Physical activity	0.012 **	0.001	0.155	0.320	0.075	0.002

B, non-standardized coefficient; SE, standard error; β, standardized coefficient. ** *p* < 0.01; *** *p* < 0.001.

**Table 5:** Multiple linear regression models used to evaluate the relation between consumption habits and intake of food and well-being.

Variable	Positive Affect (PA)			Negative Affect (NA)		
	B	SE	Β	B	SE	β
Olive oil as the main source of fat to cook	−0.033	0.060	−0.001	−0.558	0.242	−0.052
Consumption of olive oil	0.428	0.511	0.018	−0.083	0.060	−0.001
Consumption of vegetables	0.834	1.074	0.050	−0.186	0.026	−0.015
Consumption of fruit	0.169 **	0.113	0.010	−0.893	0.859	−0.069
Consumption of red meat and/or cold meat	−0.932 *	1.080	−0.055	0.497	0.830	0.039
Consumption of butter and butter by-products	0.995	1.218	0.057	−0.181 ***	0.042	−0.066
Consumption of sugary soft drinks	−0.665	1.150	−0.039	−0.288 *	0.084	−0.053
Consumption of wine	0.392	0.298	0.091	0.243	0.063	0.080
Consumption of legumes	0.344*	0.092	0.203	−0.232	0.043	−0.019
Consumption of fish or seafood	−0.341	0.099	−0.021	0.018	0.044	0.001
Consumption of industrial pastries	−0.082	0.091	−0.058	1.013 **	0.837	0.080
Consumption of nuts	0.429	0.069	0.085	0.094 *	0.061	0.086
Preferably consumption of white meat	0.034	0.606	0.040	0.179 *	0.255	0.029
Consumption of sauté with whole grains	0.402 *	0.320	0.069	−0.180 **	0.113	−0.054

B, non-standardized coefficient; SE, standard error; β, standardized coefficient. * *p* < 0.05; ** *p* < 0.01; *** *p* < 0.001. All models included age, sex, state of perceived health, physical role, mental role, and physical activity. Sauté is a traditional sauce made with tomato and onion, leek, or garlic and slow-cooked with olive oil.
